# A novel nomogram based on log odds of positive lymph nodes to predict survival for non-metastatic gallbladder adenocarcinoma after surgery

**DOI:** 10.1038/s41598-022-20933-x

**Published:** 2022-10-01

**Authors:** Shitao Jiang, Junwei Zhang, Lei Zhang, Yiyao Xu, Haitao Zhao, Xinting Sang, Xin Lu

**Affiliations:** Department of Liver Surgery, State Key Laboratory of Complex Severe and Rare Diseases, Peking Union Medical College Hospital, Peking Union Medical College, Chinese Academy of Medical Sciences, Beijing, China

**Keywords:** Oncology, Risk factors

## Abstract

The prognosis of non-metastatic gallbladder adenocarcinoma (NM-GBA) patients is affected by the status of metastatic lymph nodes. The purpose of this study was to explore the prognostic value of the log odds of positive lymph nodes (LODDS) and develop a novel nomogram to predict the overall survival in NM-GBA patients. A total of 1035 patients confirmed to have NM-GBA were selected from the Surveillance, Epidemiology, and End Results (SEER) database and further divided into training and validation cohorts. The discrimination and calibration of the nomogram were evaluated using the concordance index (C-index), the area under the time-dependent receiver operating characteristic curve (time-dependent AUC), and calibration plots. The net benefits and clinical utility of the nomogram were quantified and compared with those of the 8th edition American Joint Committee on Cancer (AJCC) Tumor-Node-Metastasis (TNM) staging system using decision curve analysis (DCA), net reclassification index (NRI), and integrated discrimination improvement (IDI). The risk stratifications of the nomogram and the TNM-staging system were compared. LODDS showed the highest accuracy in predicting OS for NM-GBA. The C-index (0.730 for the training cohort and 0.746 for the validation cohort) and the time-dependent AUC (> 0.7) indicated the satisfactory discriminative ability of the nomogram. The calibration plots showed a high degree of consistency. The DCA, NRI, and IDI indicated that the nomogram performed significantly better than the TNM-staging (P < 0.05). A novel LODDS-included nomogram was developed and validated to assist clinicians in evaluating the prognosis of NM-GBA patients.

## Introduction

Gallbladder adenocarcinoma (GBA), the sixth most common gastrointestinal cancer, is a rare disease with poor prognosis^1^. GBA is characterized by asymptomatic onset with rapid disease progression and is often diagnosed at advanced stages^2^. At present, radical surgical excision is the recommended treatment for GBA patients without distant metastasis (M0). Therefore, determining the prognostic indicators to predict outcomes for non-metastatic GBA (NM-GBA) patients after surgery will be crucial for developing possible adjuvant therapy and follow-up strategies.

Currently, survival prediction for GBA patients is based largely on the 8th edition tumor-node-metastasis (TNM) staging guidelines set forth in the American Joint Committee on Cancer (AJCC) staging system^3^. However, this staging system only incorporated factors that assess the local extension of the primary tumor, lymph node involvement and distant metastasis, ignoring other important factors such as age, gender, marital status and treatment^4–6^, which reduced the accuracy of the stratification.

Nomograms, incorporating and illustrating important prognostic factors in a visible way, have become a superior methods to accurately predict outcomes for patients with various cancers^7,8^. Previously, researchers have attempted to construct nomograms to predict the prognosis of patients with GBA^9,10^. Some novel LN schemes, such as lymph node ratio (LNR) and log odds of positive lymph node (LODDS), showed more accurate predictive ability than AJCC N status^11–13^. But most of these studies included GBA patients with distant metastases (M1 status)^14,15^. According to the 8th edition AJCC TNM-staging system, GBA patients with distant metastasis are staged as IVB and often unable to receive radical surgery, leading to extremely poor prognosis. The inclusion of M1 patients to evaluate predictive value of LNR and LODDS would result in bias. Investigating predictive capability of LNR and LODDS in NM-GBA patients is more reasonable and applicable.

To our knowledge, no previous study has examined the different LN staging/scoring systems in NM-GBA patients. Therefore, we attempted to compare the predictive value of N status, LNR, LODDS and construct a novel nomogram based on the optimal LN system and other prognostic factor to predict the prognosis of NM-GBA patients undergoing surgical treatment.

## Material and methods

### Patient selection

**Figure 1:**
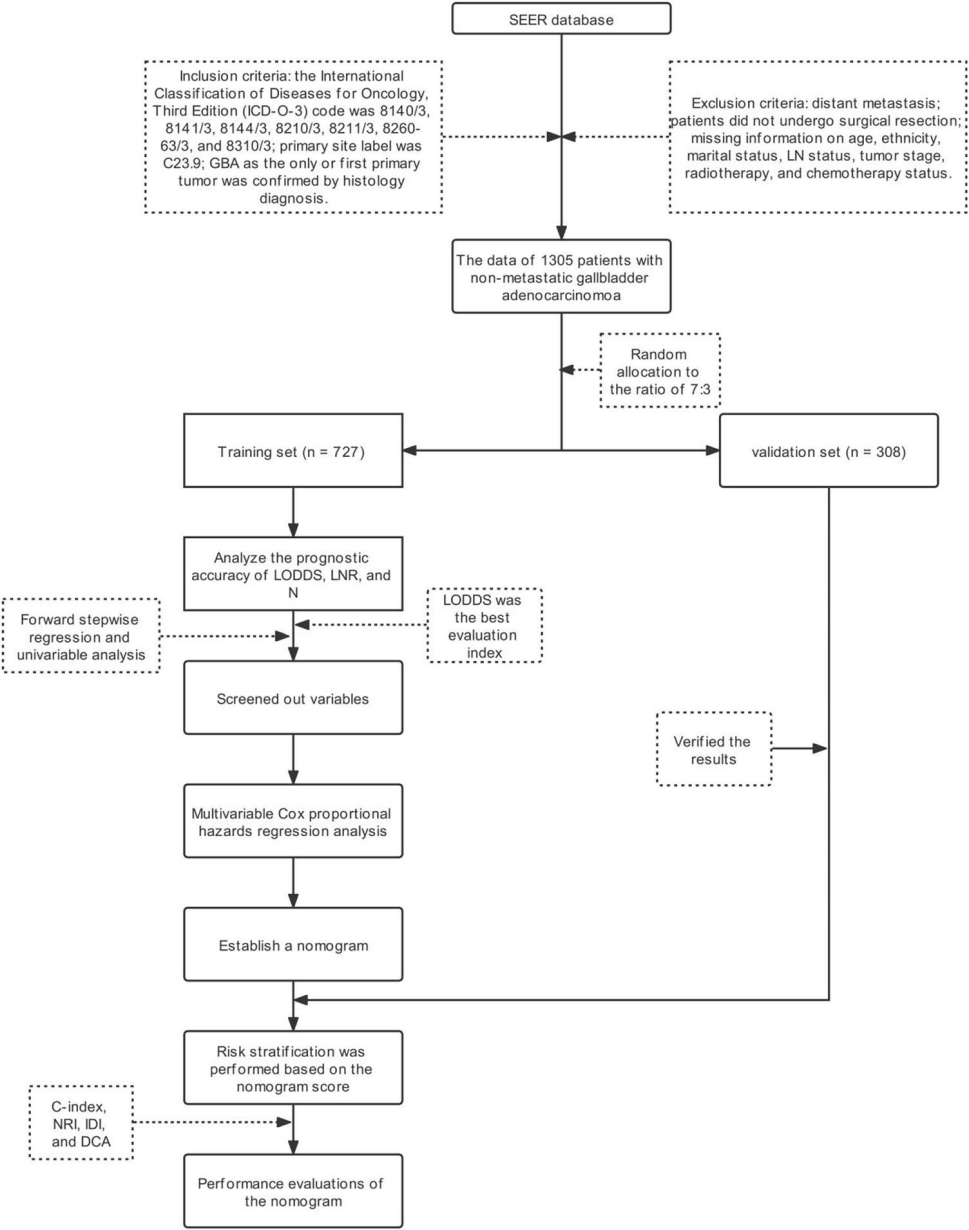
Enrollment flow chart of eligible patients in the present study.

We identified patients diagnosed with GBA from January 1, 2000, to December 31, 2018 in Surveillance, Epidemiology and End Results (SEER) database. All data analyses were performed using SEER*Stat software version 8.3.9. Inclusion criteria were as follows: the International Classification of Diseases for Oncology, Third Edition (ICD-O-3) code was 8140/3, 8141/3, 8144/3, 8210/3, 8211/3, 8260-63/3, and 8310/3^3^; primary site label was C23.9; GBA as the only or first primary tumor was confirmed by histology diagnosis. As defined by the SEER database, M1 meant that distant metastases occurred at the time of initial diagnosis. The exclusion criteria were as follows: distant metastasis; patients did not undergo surgical resection; missing information on age, ethnicity, marital status, LN status, tumor stage, radiotherapy and chemotherapy status. Our detailed workflow was shown in Fig. 1.

### Cohort definition and variable screening

Based on the inclusion and exclusion criteria, 1035 patients were included in the discovery cohort. By using the "createDataPartiton" function in R (version 4.1.0), the discovery cohort was divided into training and validation cohorts with a ratio of 7:3. Training cohort was used to build models, whereas validation cohort was used to compare models. A total of 15 different clinical variables were included. Staging was reclassified using best available data according to AJCC eighth edition criteria. In this study, LNR was defined as the ratio of the number of positive lymph nodes (NPLN) to the number of resected lymph nodes (NRLN). LODDS was calculated by log[(NPLN + 0.05)/(NRLN-NPLN + 0.05)].

Two continuous variables (LNR and LODDS) were all trichotomized via the X-tile software (version 3.6.1; Yale University, New Haven, CT, USA) based on the maximal log-rank chi-square value, which represented the greatest group difference in outcome probability^16^. In our study, LNR was categorized into LNR1 (range, 0 to 0), LNR2 (range, 0.04 to 0.88), and LNR3 (range, 0.89 to 1.0). LODDS was classified into LODDS1 (range, − 2.9 to − 1.61), LODDS2 (range, − 1.36 to 0.83), and LODDS3 (range, 0.89 to 2.42). Performance of LODDS, LNR, and N status were evaluated from multiple dimensions. The likelihood-ratio (LR) test was used to assess homogeneity between groups. Akaike Information Criterion (AIC) and bayesian information criterion (BIC) were applied to test goodness of fit^17^. Harrell concordance index (C-index) was calculated to assess the accuracy of prediction. Univariate and multivariate Cox proportional hazards regression models were utilized to detect independent prognostic factors.

### Statistical analysis

The multivariate model used a forward stepwise variable selection procedure based on AIC. Model discrimination was assessed using the time-dependent area under the ROC curves (AUC) and the C-index, and calibration was measured using calibration plots. Based on previous research, C-index and AUC value greater than 0.7 indicated that the nomogram had good predictive power. Using the AJCC TNM staging system as a reference, integrated discrimination improvement (IDI) and net reclassification improvement (NRI)^18,19^ were calculated to assess the improvement of clinical benefits; Decision curve analysis (DCA)^20^ was used to evaluate the clinical utility of the nomogram. Survival analysis was performed using Kaplan–Meier curves and Cox proportional hazard models to compare the risk stratification effect of the nomogram with the AJCC staging system.

The primary endpoint was overall survival (OS), defined as the time from tumor excision to death or the last follow-up. Multicollinearity was checked using the variance inflation factor (VIF). Variables with VIF > 4 were removed. Categorical variables were compared by the Chi-square test, while the Kruskal–Wallis test was utilized to compare continuous data across groups. All tests were two-tailed, and P values < 0.05 were considered statistically significant. For all statistical analyses, R (version 4.1.0) was performed.

### Ethical statement

This study was conducted in accordance with the ethical guidelines mandated by the Declaration of Helsinki. With use of publicly available data, this study was considered exempt from review and thus no patient written informed consent was needed. We signed the 'Surveillance, Epidemiology, and End Results Program Data Use Agreement' for accessing the SEER database legally. The author ST Jiang has gotten access to the SEER database (accession number: 16609-Nov2020).

## Results

### Patient characteristics

A total of 1035 patients with NM-GBA were included and randomly assigned into a training (n = 727) and validation (n = 308) cohorts. Cumulative survival in the whole cohort was 73.5% at 1 year, 44.2% at 3 year, and 33.9% at 5 year. Baseline characteristics of the patients included in the training and validation sets were balanced (Table 1).

**Table 1:** Demographic and clinical characteristics of patients with NM-GBA.

Characteristic	Item	Overall	Training cohort	Validation cohort	*P* value
**Total, n**		1035	727	308	
**Sex, n (%)**	Female	721 (69.7%)	515 (70.8%)	206 (66.9%)	0.233
	Male	314 (30.3%)	212 (29.2%)	102 (33.1%)	
**Race, n (%)**	White	791 (76.4%)	556 (76.5%)	235 (76.3%)	0.301
	Black	112 (10.8%)	84 (11.6%)	28 (9.1%)	
	Other	132 (12.8%)	87 (12%)	45 (14.6%)	
**Grade, n (%)**	G1	153 (14.8%)	110 (15.1%)	43 (14%)	0.805
	G2	493 (47.6%)	339 (46.6%)	154 (50%)	
	G3	375 (36.2%)	268 (36.9%)	107 (34.7%)	
	G4	14 (1.4%)	10 (1.4%)	4 (1.3%)	
**T, n (%)**	T1	118 (11.4%)	92 (12.7%)	26 (8.4%)	0.162
	T2	533 (51.5%)	374 (51.4%)	159 (51.6%)	
	T3	352 (34%)	237 (32.6%)	115 (37.3%)	
	T4	32 (3.1%)	24 (3.3%)	8 (2.6%)	
**N, n (%)**	N0	560 (54.1%)	393 (54.1%)	167 (54.2%)	0.816
	N1	437 (42.2%)	309 (42.5%)	128 (41.6%)	
	N2	38 (3.7%)	25 (3.4%)	13 (4.2%)	
**Stage, n (%)**	Stage I	107 (10.3%)	81 (11.1%)	26 (8.4%)	0.596
	Stage II	295 (28.5%)	207 (28.5%)	88 (28.6%)	
	Stage III	567 (54.8%)	392 (53.9%)	175 (56.8%)	
	Stage IV	66 (6.4%)	47 (6.5%)	19 (6.2%)	
**Radiation, n (%)**	None	765 (73.9%)	539 (74.1%)	226 (73.4%)	0.858
	Yes	270 (26.1%)	188 (25.9%)	82 (26.6%)	
**Chemotherapy, n (%)**	None	613 (59.2%)	438 (60.2%)	175 (56.8%)	0.338
	Yes	422 (40.8%)	289 (39.8%)	133 (43.2%)	
**Marital, n (%)**	Married	564 (54.5%)	397 (54.6%)	167 (54.2%)	0.211
	Single	140 (13.5%)	92 (12.7%)	48 (15.6%)	
	Separated	112 (10.8%)	87 (12%)	25 (8.1%)	
	Widowed	219 (21.2%)	151 (20.8%)	68 (22.1%)	
**NRLN, median (IQR)**		2 (1, 5)	2 (1, 4)	2 (1, 5)	0.255
**NPLN, median (IQR)**		0 (0, 1)	0 (0, 1)	0 (0, 1)	0.880
**LNR, median (IQR)**		0 (0, 0.8)	0 (0, 1)	0 (0, 0.67)	0.722
**LODDS, median (IQR)**		-1.32 (-1.613, 0.591)	-1.32 (-1.61, 1.32)	-1.32 (-1.79, 0.29)	0.490
**Size (mm), median (IQR)**		30 (20, 42)	30 (20, 40)	30 (20, 45)	0.742
**Age (years), median (IQR)**		68 (60, 77)	69 (60, 78)	68 (59.75, 76)	0.382
**Survival, median (IQR)**		28 (11, 56)	28 (11, 56)	30 (11, 56.25)	0.984

*NRLN* number of resected lymph nodes, *NPLN* number of positive lymph nodes, *LNR* lymph node ratio, *LODDS* log odds of positive lymph nodes, *IQR* interquartile range.

### Variables screening

The C-index, LR, AIC and BIC were used to compare the predictive abilities of N stage, LNR, and LODDS. The LODDS system showed the highest C-index and LR test as well as the lowest AIC and BIC, which indicated that the superior performance of LODDS (C-index: 0.648, LR test: 133.4, AIC: 5863.572, BIC: 5872.009) over LNR (C-index: 0.637, LR test: 128, AIC: 5868.957, BIC: 5877.395) and N status (C-index: 0.622, LR test: 104.1, AIC: 5892.882, BIC: 5901.309) in terms of predicting OS for NM-GBA. To determine the optimal model, we performed forward stepwise regression with the LODDS and other ten significant modules. Finally, age, sex, chemotherapy, stage, grade, LODDS, and size were included. The univariate Cox regression analysis revealed that 9 variables (age, sex, stage, grade, LODDS, size, race, T, and marital) were significantly associated with OS in the training cohort. In multivariate Cox regression analysis, age, sex, chemotherapy, stage, grade, LODDS, and size remained as independent prognostic factors (Table 2).

**Table 2:** Univariate and multivariate Cox analyses on variables for the prediction of overall survival of NM-GBA patients.

Variable	Univariate analysis			Multivariate analysis	
	HR (95% CI)	P value		HR (95% CI)	P value
**Sex**					
Female	Reference				
Male	1.275 (1.055–1.541)	0.012		1.414 (1.153–1.735)	< 0.001
**Race**					
White	Reference				
Black	0.803 (0.606–1.066)	0.129		1.013 (0.757–1.355)	0.933
Other	0.750 (0.563–0.999)	0.049		0.963 (0.715–1.298)	0.806
**Grade**					
G1	Reference				
G2	1.539 (1.155–2.051)	0.003		1.320 (0.978–1.780)	0.070
G3	1.982 (1.479–2.657)	< 0.001		1.350 (0.987–1.847)	0.060
G4	3.075 (1.466–6.448)	0.003		2.250 (1.056–4.796)	0.036
**T**					
T1	Reference				
T2	1.286 (0.937–1.764)	0.119		0.604 (0.302–1.210)	0.155
T3	2.722 (1.973–3.754)	< 0.001		1.123 (0.559–2.253)	0.745
T4	4.883 (2.901–8.221)	< 0.001		0.944 (0.381–2.339)	0.901
**Stage**					
Stage I	Reference				
Stage II	1.046 (0.719–1.522)	0.813		1.635 (0.740–3.612)	0.224
Stage III	2.781 (1.977–3.913)	< 0.001		2.042 (0.937–4.452)	0.072
Stage IV	4.855 (3.092–7.622)	< 0.001		4.271 (1.745–10.453)	0.001
**Radiation**					
None	Reference				
Yes	0.960 (0.788–1.169)	0.684		0.813 (0.636–1.039)	0.098
**Chemotherapy**					
None	Reference				
Yes	0.954 (0.798–1.141)	0.605		0.770 (0.606–0.979)	0.033
**LODDS**					
LODDS1	Reference				
LODDS2	1.899 (1.511–2.387)	< 0.001		1.792 (1.405–2.286)	< 0.001
LODDS3	4.244 (3.313–5.436)	< 0.001		3.221 (2.373–4.371)	< 0.001
**Size (mm)**					
1–13	Reference				
14–56	2.040 (1.521–2.737)	< 0.001		1.487 (1.091–2.028)	0.012
57–160	3.446 (2.412–4.923)	< 0.001		2.254 (1.540–3.297)	< 0.001
**Age (years)**					
29–72	Reference				
73–82	1.712 (1.402–2.090)	< 0.001		1.601 (1.289–1.988)	< 0.001
83–100	2.630 (2.043–3.384)	< 0.001		1.908 (1.421–2.562)	< 0.001
**Marital**					
Married	Reference				
Single	0.954 (0.721–1.263)	0.744		1.193 (0.889–1.599)	0.240
Separated	1.000 (0.751–1.331)	0.997		1.055 (0.778–1.429)	0.731
Widowed	1.498 (1.208–1.858)	< 0.001		1.222 (0.958–1.558)	0.106

*HR* hazard ratio, *CI* confidence interval, *LODDS* log odds of positive lymph nodes.

### Nomogram construction and validation

**Figure 2:**
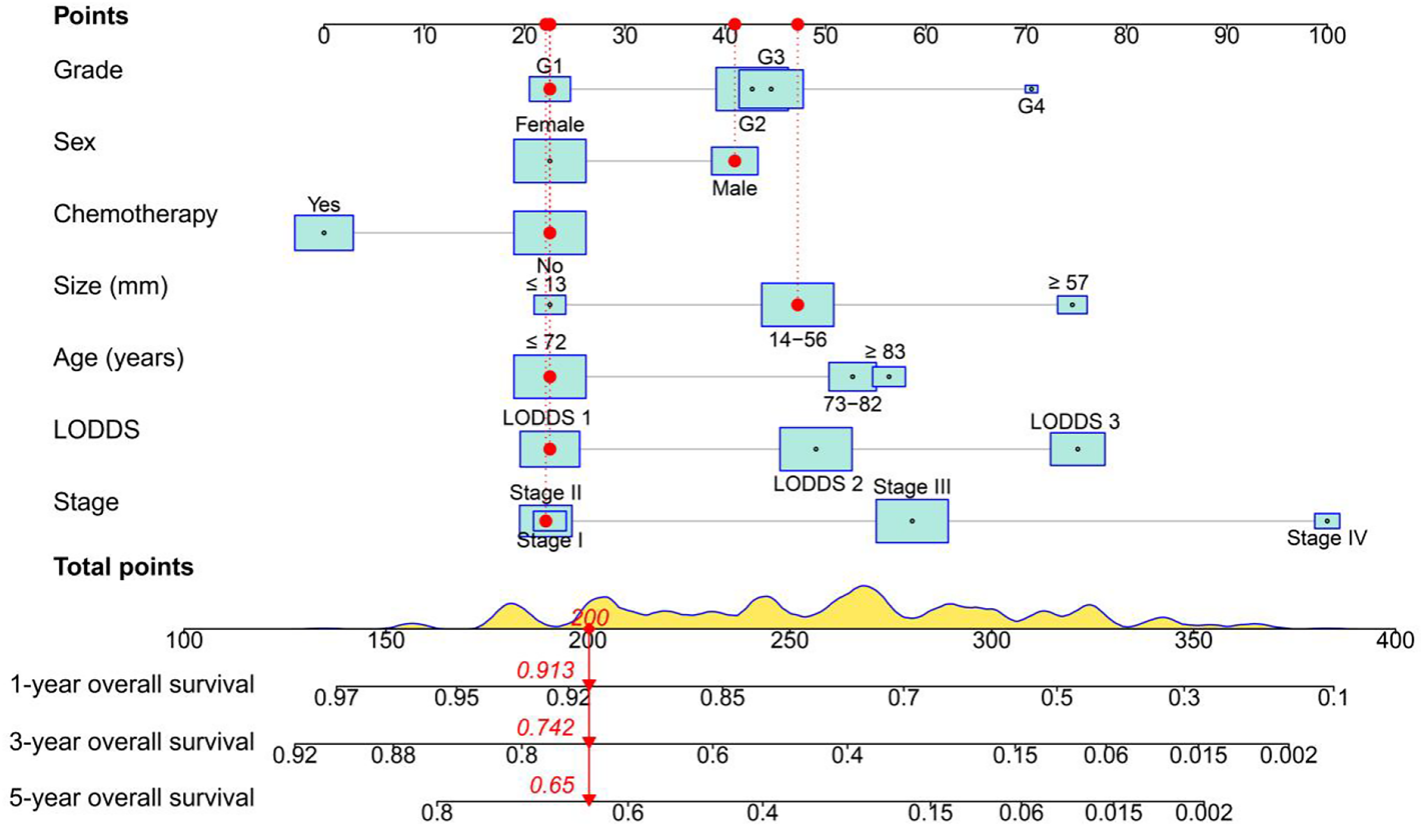
A constructed nomogram for prognostic prediction of a patient with NM-GBA. For category variables, their distributions were reflected by the size of the box (to view boxes of the stage, the smaller one represents stage I and the bigger one represents stage II). The density plot of total points shows the distribution. The patient, a 29-year-old man, had a tumor of 24 mm, G1, stage II, LODDS 1 (−2.479), and did not receive chemotherapy. To use the nomogram, the specific points (black dots) of individual patients were located on each variable axis. Red lines and dots were drawn upward to determine the points received by each variable; the sum (200) of these points was located on the Total Points axis, and a line was drawn downward to the survival axes to determine the probability of 1-year (91.3%), 3-year (74.2%) and 5-year (65.0%) overall survival. *NM-GBA* non-metastatic gallbladder adenocarcinoma, *LODDS* log odds of positive lymph nodes.

The new prognostic model for NM-GBA based on these variables was constructed. A nomogram displaying the predictive variables and corresponding point scales was shown in Fig. 2. The nomogram estimated the survival probability for a patient based on a total score which is calculated by the addition of zero to 100 points for every individual predictor. Most patients in the present study had total risk points ranging from 137 to 384.

**Figure 3:**
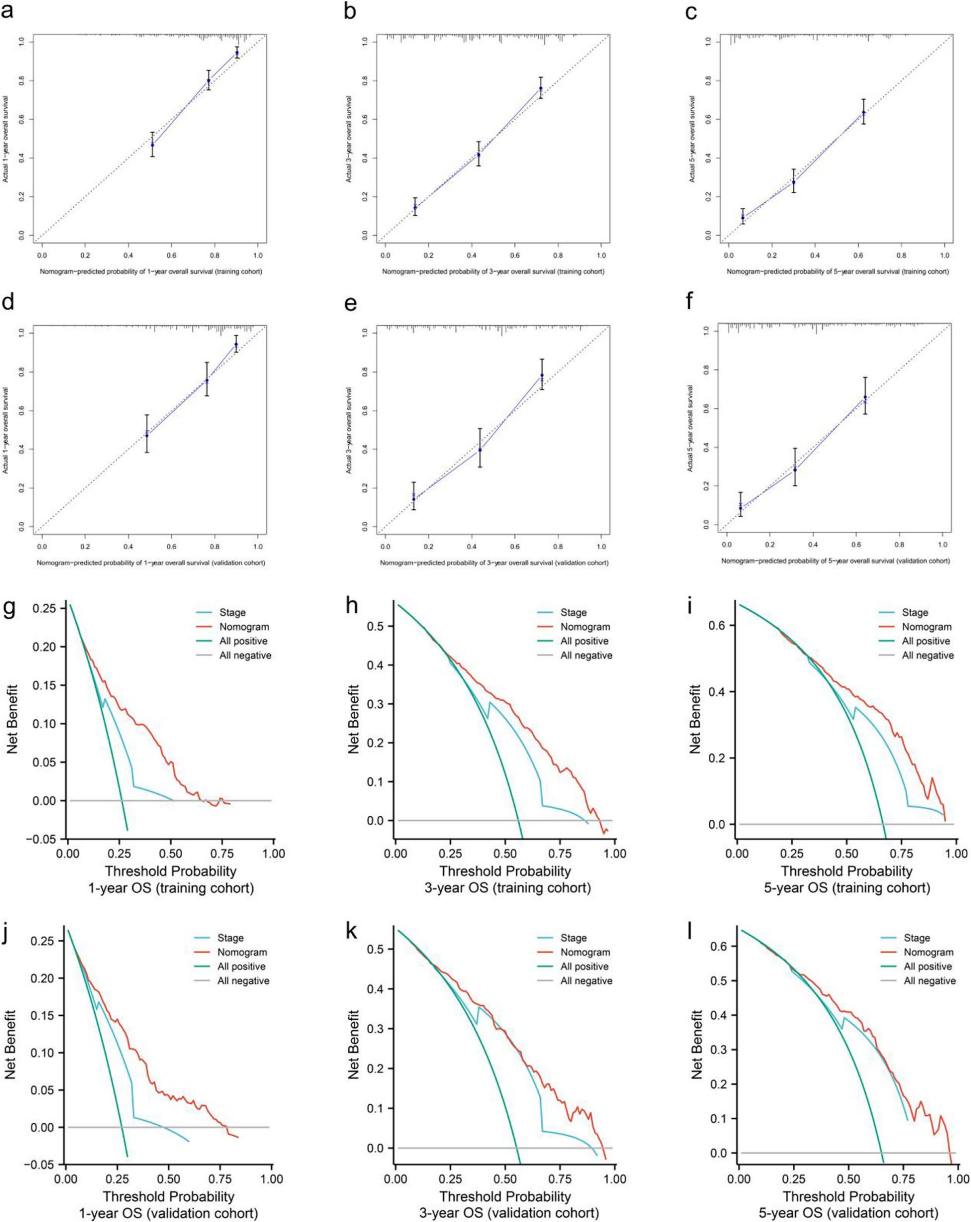
Calibration curves and decision curve analysis of the nomogram for the survival prediction of patients with NM-GBA. (**a–f**) Calibration curves of 1-year, 3-year, and 5-year OS in the training and validation cohorts. (**g–l**) 1-year, 3-year, and 5-year survival benefit of in the training and validation cohorts. *NM-GBA* non-metastatic gallbladder adenocarcinoma, *OS* overall survival.

The C-index of the nomogram in the training and validation cohorts were 0.730 (0.708–0.752) and 0.746 (0.715–0.777), respectively. The time-dependent AUC was > 0.7 for the prediction of OS within 1, 3, and 5 years in both the training and validation cohorts (training cohort: 1-year AUC = 0.802 (0.766–0.838), 3-year AUC = 0.803 (0.770–0.835), 5-year AUC = 0.794 (0.756–0.832); validation cohort: 1-year AUC = 0.784 (0.749–0.819), 3-year AUC = 0.784 (0.751–0.817), 5-year AUC = 0.786 (0.751–0.821)), indicating favorable discrimination of the predictive model. Calibration plots demonstrated good agreement between nomogram-predicted and observed events (Fig. 3a–f). The high calibration and discrimination performance of the nomogram was confirmed in the validation cohort.

### Clinical value of the nomogram compared with the AJCC 8th edition TNM-staging system

In order to evaluate the accuracy of change in risk classification, we calculated the NRI, IDI, C-index, and AUC of the nomogram and the AJCC 8th edition TNM-staging system. When testing the nomogram in training cohort, the NRI for the 1-, 3-, and 5-year OS were 0.648 (95% CI = 0.532–0.862), 0.625 (95% CI = 0.480–0.757), and 0.589 (95% CI = 0.434–0.739), the IDI for the 1-, 3-, and 5-year OS were 0.073 (95% CI = 0.058–0.088, P < 0.001), 0.102 (95% CI = 0.072–0.132, P < 0.001), and 0.099 (95% CI = 0.081–0.117, P < 0.001), and the change of C-index was 0.191 (95% CI = 0.169–0.213, P < 0.001). In addition, we compared the AUC values of different prediction models. The results showed that the AUC values for the nomogram were higher than the AJCC 8th edition TNM-staging system (Table 3).

**Table 3:** NRI, IDI, C-index, and AUC of the nomogram and the AJCC 8th edition TNM-staging system in survival prediction for NM-GBA patients.

Index	Training cohort			Validation cohort		
	Estimate	95% CI	*P* value	Estimate	95% CI	*P* value
**NRI (vs. stage)**						
1-year OS	0.648	0.532–0.862		0.714	0.411–0.950	
3-year OS	0.625	0.480–0.757		0.495	0.343–0.791	
5-year OS	0.589	0.434–0.739		0.486	0.301–0.799	
**IDI (vs. stage)**						
1-year OS	0.073	0.058–0.088	< 0.001	0.078	0.054–0.102	< 0.001
3-year OS	0.102	0.072–0.132	< 0.001	0.102	0.073–0.131	< 0.001
5-year OS	0.099	0.081–0.117	< 0.001	0.097	0.068–0.126	< 0.001
**C-index**						
Nomogram	0.730	0.708–0.752		0.746	0.717–0.775	
AJCC stage	0.639	0.617–0.661		0.660	0.631–0.689	
Change	0.191	0.169–0.213	< 0.001	0.186	0.157–0.215	< 0.001
**AUC**						
Nomogram 1 Y	0.802	0.766–0.838		0.784	0.749–0.819	
AJCC stage 1 Y	0.666	0.629–0.704		0.694	0.645–0.744	
Nomogram 3 Y	0.803	0.770–0.835		0.784	0.751–0.817	
AJCC stage 3 Y	0.704	0.669–0.739		0.741	0.690–0.792	
Nomogram 5 Y	0.794	0.756–0.832		0.786	0.751–0.821	
AJCC stage 5 Y	0.701	0.660–0.742		0.742	0.682–0.802	

*NRI* net reclassification improvement, *IDI* integrated discrimination improvement, *CI* confidence interval, *OS* overall survival, *AJCC* American Joint Committee on Cancer, *AUC* area under the curve, *Y* year.

This results indicated that the prognostic performance of the new-built model was superior than that of the traditional AJCC TNM staging system. DCA showed the nomogram could better predict the 1-year, 3-year, and 5-year OS in NM-GBA patients. Compared to the AJCC 8th edition system, the nomogram added more net benefits to almost all threshold probabilities in the training and validation cohorts (Fig. 3g–l).

### Clinical risk stratification of NM-GBA patients based on nomogram score

**Figure 4:**
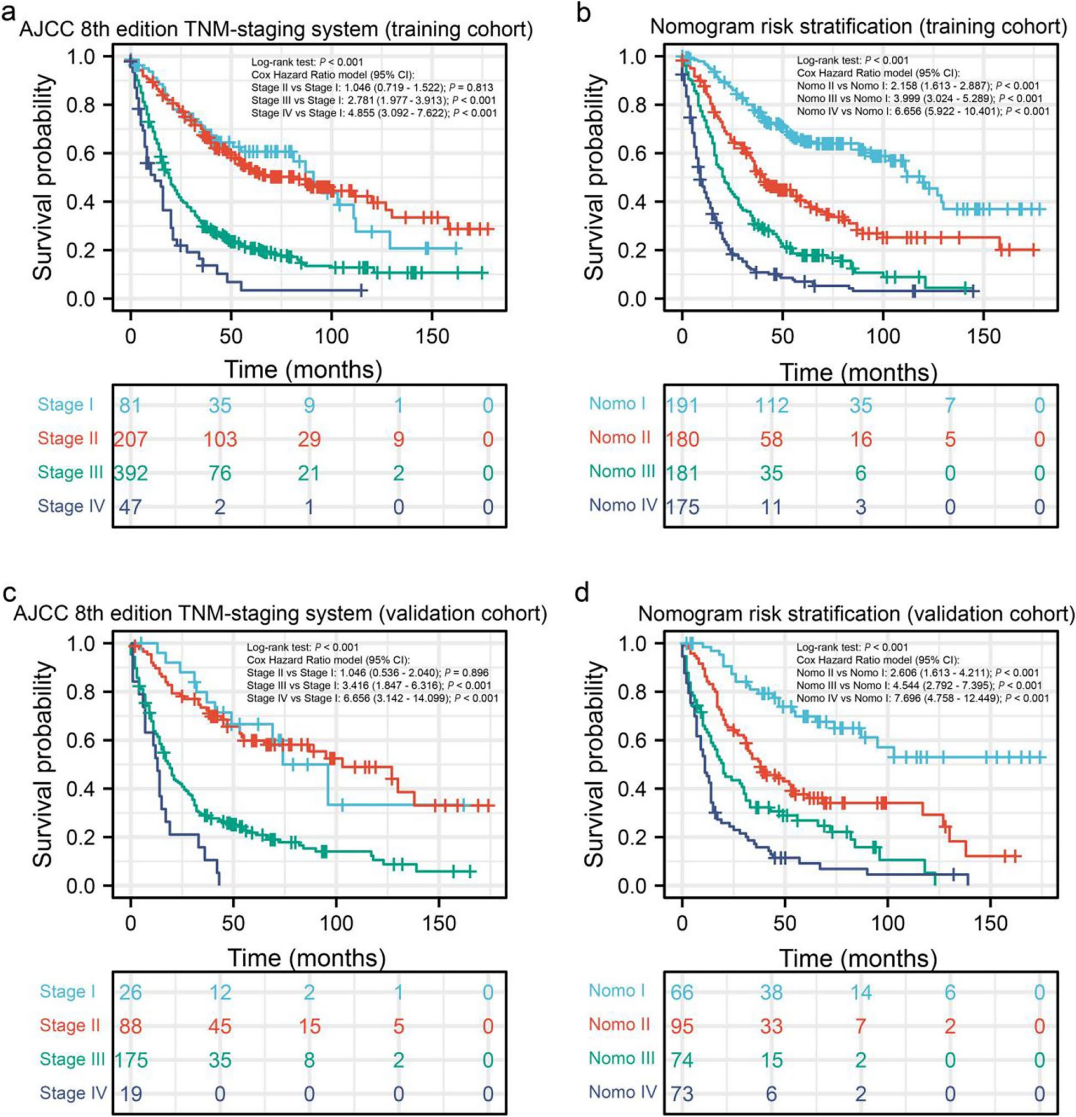
Kaplan–Meier overall survival curves of patients with NM-GBA at different stages or with different risks stratified by the nomogram. (**a**) NM-GBA patients in the training cohort at different stages were classified according to the AJCC 8th edition TNM-staging system. (**b**) NM-GBA patients in the training cohort at different risks were stratified according to the nomogram. (**c**) NM-GBA patients in the validation cohort at different stages were classified according to the AJCC 8th edition TNM-staging system. (**d**) NM-GBA patients in the validation cohort at different stages were stratified according to the nomogram. *NM-GBA* non-metastatic gallbladder adenocarcinoma; *AJCC* American Joint Committee on Cancer, *TNM* tumor, node, metastasis.

We finally stratified the risk of patients in the training and validation cohorts based on the total score calculated by the nomogram. Patients can be divided into four groups: nomo 1 (total score < 223), nomo 2 (223 ≤ total points < 265), nomo 3 (265 ≤ total points < 296), and nomo 4 (total points ≥ 296). As shown in Fig. 4, the differences between the Kaplan–Meier survival curves of all four groups of patients in Nomo I-IV were statistically significant (P < 0.05). However, the differences between Stage II and I were not statistically significant. In addition, after risk stratification according to the AJCC stage, there was a significant intersection of Kaplan–Meier survival curves for patients in different stages. The Kaplan–Meier survival curves showed significant discrimination among the four risk groups of the Nomo staging system. However, the AJCC 8th edition staging system performed limited ability to identify patients at high and low risk in training and validation cohorts (Fig. 4).

## Discussion

This study found that LODDS was the best independent indicator to describe the relationship between LN metastasis status and prognosis in patients with NM-GBA who underwent surgical treatment. The nomogram constructed using LODDS and six other variables (grade, sex, chemotherapy, size, age, and stage) could accurately predict the prognosis at 1-, 3-, and 5-years in patients with NM-GBA. Its prediction accuracy was higher than that of AJCC 8th edition TNM-staging system.

Currently, adjuvant therapy after surgery for patients with GBC is still controversial. Three expert groups had provided consensus guidelines for the adjuvant treatment of GBC after resection. The American Society of Clinical Oncology (ASCO) clinical practice guideline recommended that all patients with GBC who undergo radical resection should receive six months of adjuvant capecitabine^21^. However, the National Comprehensive Cancer Network (NCCN) guidelines recommended options including observation, fluoropyrimidine-based radiotherapy, fluoropyrimidine-based chemotherapy, or gemcitabine-based chemotherapy for patients with stage T1b or greater, negative margins, and negative lymph nodes. Observation only was recommended for patients with post-excisional (incidentally discovered) stage T1a lesions with negative margins^22^. For patients with positive margins or positive lymph nodes, options included fluoropyrimidine-based chemotherapy, gemcitabine-based chemotherapy, fluoropyrimidine-based radiotherapy, or combination therapy^22^. 2016 European Society of Medical Oncology (ESMO) consensus guidelines recommended that adjuvant therapy be considered for resected GBC only after a risk–benefit assessment^23^. The nomogram we developed provided an accurate and reliable risk stratification method for clinical practice. For low-risk patients, intensive observation might be a reasonable strategy, which might help patients avoid harm caused by excessive medical treatment. For high-risk patients, such as Nomo III or Nomo IV, aggressive postoperative adjuvant chemotherapy or radio-chemotherapy might be necessary. Previous studies have shown that it might improve the prognosis of patients. Therefore, we believe that risk stratification based on the nomogram could assist clinicians to identify patients for more active follow-up and adjuvant postoperative treatment.

Since a report in 2015 by Amini N et al., a total of 7 articles have analyzed the prognostic value of LODDS in gallbladder cancer (GBC). Amini N et al. firstly analyzed 1124 patients with GBA in the SEER database from 2004 to 2010. They found that LODDS performed better than AJCC N status and LNR when the NRLN ≥ 4. In a subsequent study, they confirmed the predictive advantage of LODDS in a multicenter cohort of 214 patients with GBA^24^. In addition, Lee W et al.'s analysis of 398 patients with GBC showed that LODDS was the most appropriate indicator when NRLN ≥ 6^25^. Chen C et al. reached a different conclusion by analyzing a multicenter cohort of 226 patients with GBC. They used three different modeling methods to demonstrate that NPLN was the best LN system among various descriptions of LN status. However, they did not propose a new model based on LODDS to predict the prognosis of GBC patients. A total of three previous studies have constructed a prediction model including LODDS to predict the OS of patients with GBC. In their study, the C-index in the training set was 0.735–0.752, and the C-index in validation set was 0.719–0.752^14,15,26^. Table 4 summarized the major studies that developed LODDS-based predictive nomograms in GBC management.

**Table 4:** Comparison of major studies that developed predictive nomograms included LODDS in GBC.

References	Type of study, sample size	Purpose of study	Parameters included	C-index (95% CI) of nomogram in validation set
Xiao et al., 2019^23^	SEER database analysis, n = 1321	To establish a prognostic model including LODDS of GBC patients after resection	Age, grade, size, LODDS, AJCC 8th edition T, AJCC 8th edition M	0.752 (0.720–0.768)
Li et al., 2020^15^	SEER database analysis, n = 1612	To establish a prognostic model including LODDS of GBA patients over 45 years after resection	Age, grade, size, LODDS, AJCC 8th edition T, AJCC 8th edition M, marital status	0.740 (0.721–0.759)
Yuan et al., 2021^14^	SEER database analysis and single-center study, n = 856	To establish a prognostic model including LODDS of patients with T3 and T4 GBC after resection	Age, grade, LODDS, AJCC 8th edition M, chemotherapy, radiotherapy	0.719 (0.707–0.713)
Current study	SEER database analysis, n = 1035	To establish a prognostic model including LODDS of patients with non-metastatic GBA after resection	Age, grade, size, sex, LODDS, AJCC 8th edition stage, chemotherapy	0.746 (0.717–0.775)

*LODDS* log odds of positive lymph nodes, *GBC* gallbladder cancer, *GBA* gallbladder adenocarcinoma, *CI* confidence interval, *SEER* surveillance, epidemiology and end results.

Although all of the above nomograms perfomed better predictive ability than AJCC 8th edition TNM-staging system, they included patients with distant metastases (M1) in the study cohort. The study by Xiao et al. aimed to assess the prognostic value of different lymph node staging systems and to develop a prognostic prediction model after curative surgery^26^. However, in their study, patients with stage M1 accounted for 13.5%. In fact, M1 status represented that patients showed distant metastasis at the time of evaluation. These patients almostly lost the opportunity to undergo radical surgery and the extent of LN dissection varies widely. Therefore, it is more reasonable to avoid the inclusion of these patients when evaluating the predictive power of the LN staging system^27^. Instead, the present study only included patients with NM-GBA to more accurately assess the predictive power of LODDS. In addition, we also noticed that in previous studies, researchers did not pay attention to the collinearity of variables, which would cause the bias of the model. Therefore, in this study, we not only included as many variables as possible but also used the stepwise regression method to screen independent predictors, and calculated the collinearity of variables, thus ensuring the nomogram more stable.

A total of seven variables (age, sex, chemotherapy, stage, grade, LODDS, and size) were incorporated to construct the nomogram. In accordance with previous studies, advanced age, male gender, high tumor stage, high tumor grade, and large tumor size were independent prognostic factors associated with OS of GBC patients^28–30^. By analyzing 1137 patients with GBC in the SEER-Medicare database, Samuel J. Wang et al. found that some GBC patients could benefit from adjuvant chemotherapy^31^. Interestingly, in this study, chemotherapy was not significantly associated with prognosis in univariate Cox regression analysis, but it showed as independent prognostic factor in multivariate Cox regression analysis and stepwise regression analysis. The possible reason was that there might be a certain correlation between chemotherapy and other confounding factors. In univariate Cox regression analysis, the true effect of this factor was covered by the effect of other confounding factors. Only when the effect of other factors were eliminated through multivariate Cox regression analysis can the prognostic effect of chemotherapy be revealed^32^.

We divided patients into nomo1-4 groups according to their nomogram total points. The Kaplan–Meier method and Cox hazard ratio model demonstrated significant differences in OS among the four risk groups with better discrimination than the conventional staging system. In particular, the nomogram had greater ability to recognize the high-risk population than the conventional staging system. Due to the poor outcomes, particular attention should be paid to patients with total points ≥ 296.

There were limitations to our study. Firstly, this study was a retrospective study based on the SEER database. Although we performed internal validation to confirm the nomogram's excellent predictive power, external validation based on multicenter cohorts would be needed in future studies. Secondly, the cut-off values of variables included in this study were obtained by X-tile software, which might not be applicable to other research. The appropriate cut-off values need to be explored by larger samples. In addition, because the SEER database only recorded cursory information on the surgical approach and chemotherapy, it was not easy to define the two rigorously in our manuscript, which might reduce the reproducibility of this study. Finally, The SEER database only included population from each state in the United States, so whether the prediction model established thus could be directly applied to studies on different population backgrounds needed further verification.

## Conclusion

In conclusion, the LODDS-based nomogram has a significantly higher accuracy than the AJCC 8th edition TNM-staging to predict OS for NM-GBA patients. The novel staging system based on the nomogram score can intuitively and quantitively stratify NM-GBA patients after surgical treatment and further assist to design postoperative adjuvant chemotherapy.

## Data Availability

All data included in this article for analysis can be found at https://seer.cancer.gov/. The data that support the findings of this study are available from the SEER database but restrictions apply to the availability of these data, which were used under license for the current research, and so are not publicly available. Data are, however, available from the authors upon reasonable request and with permission of the SEER database.
